# Does peer learning or higher levels of e-learning improve learning abilities? A randomized controlled trial

**DOI:** 10.3402/meo.v18i0.21877

**Published:** 2013-11-13

**Authors:** Bjarne Skjødt Worm, Kenneth Jensen

**Affiliations:** Department of Anaesthesia and Intensive Care, Copenhagen University Hospital Bispebjerg, Copenhagen, Denmark

**Keywords:** level, e-learning, learning, teaching

## Abstract

**Background and aims:**

The fast development of e-learning and social forums demands us to update our understanding of e-learning and peer learning. We aimed to investigate if higher, pre-defined levels of e-learning or social interaction in web forums improved students’ learning ability.

**Methods:**

One hundred and twenty Danish medical students were randomized to six groups all with 20 students (eCases level 1, eCases level 2, eCases level 2+, eTextbook level 1, eTextbook level 2, and eTextbook level 2+). All students participated in a pre-test, Group 1 participated in an interactive case-based e-learning program, while Group 2 was presented with textbook material electronically. The 2+ groups were able to discuss the material between themselves in a web forum. The subject was head injury and associated treatment and observation guidelines in the emergency room. Following the e-learning, all students completed a post-test. Pre- and post-tests both consisted of 25 questions randomly chosen from a pool of 50 different questions.

**Results:**

All students concluded the study with comparable pre-test results. Students at Level 2 (in both groups) improved statistically significant compared to students at level 1 (*p*>0.05). There was no statistically significant difference between level 2 and level 2+. However, level 2+ was associated with statistically significant greater student's satisfaction than the rest of the students (*p*>0.05).

**Conclusions:**

This study applies a new way of comparing different types of e-learning using a pre-defined level division and the possibility of peer learning. Our findings show that higher levels of e-learning does in fact provide better results when compared with the same type of e-learning at lower levels.

While social interaction in web forums increase student satisfaction, learning ability does not seem to change. Both findings are relevant when designing new e-learning materials.

## Introduction

e-Learning is often defined as a learning method employing electronic media and technology with or without the physical presence of a classroom and a teacher. It includes a wide range of learning platforms such as the Internet, computer programs, and multimedia content by other digital delivery methods. Since many examples of e-learning discard the physical interaction between pupil and teacher, e-learning requires some basic prerequisites on behalf of both teacher and pupil regarding the technology used for these multimedia interfaces, a commitment to carry out the full learning module by the pupil alone, and a method of controlling the learning process for the teacher. Historically, many learning methods have been used, but in recent years, e-learning has been increasingly integrated into medical education with the expansion and dissemination of digital platforms for everyday use (1). These educational applications are being developed for both pre- and post-graduate training (examples are eFront, Moodle, Dokeos, Claroline, Ilias) and used at Universities as part of their curriculum. As mentioned previously, e-learning differs from former educational methods in the shift from teaching to learning, in which the student is required to actively search knowledge instead of being a passive recipient (2).

### Quality of e-learning studies

A major issue when discussing the value of e-learning is the potential difference in material quality, communication skills, and digital setup, making comparisons between learning methods uneven and hard to quantify. The highly variable description methodology used when describing e-learning demonstrates that the reliability of data regarding the efficacy of e-learning on both direct and long-term effects may be misleading. The quality of many studies also varies, with a control group either lacking or not clearly defined (3). Given these caveats, studies show that e-learning performs statistically significant better on several success parameters than no educational method, but that it often performs equally well to traditional classroom teaching (3). These broad outlines of results do not adequately reflect the true place of e-learning within the spectrum of medical learning methods since the nature of the learning material may be widely different between studies.

### Standardization of e-learning material using levels

A systematic description of e-learning content is very much needed, but formerly used scales are becoming insufficient with the advent of recent technological developments (1, 4–6). We suggest a new taxonomy where we divide e-learning into three different *types* and three different *levels*. *Type* corresponds to the level of Bloom's taxonomy (7) targeted (this could be presentations, scenarios, or games/simulations), while *level* refers to the multimedia development level. Multimedia *level 1* includes text, basic images, audio, simple interactivities for content presentation and a template layout used through all e-learning pages. Level 2 adds video, simple animations, and variations on the presented e-learning pages (Table 1). e-Learning material may thus be divided into nine different categories. Learning objectives include recall, analysis, and problem solving, each of which may be achieved to different degrees for each learning type and level (8–11).

**Table 1:** Proposed *levels* in modern e-learning

Course level	
Level 1	Text, images, audio, simple interactivities for content presentation. Template layout
Level 2	Level 1+video and simple animations. Fewer pages with template layout
Level 3	Complex animation, high-fidelity/3D graphics, complex multilevel, and multivariable interaction

The development of e-learning materials is also a topic to consider and focus should be directed on both clinical relevance, pedagogical principles, and well-developed e-learning platforms (12).

### E-peer learning

In addition to these levels of e-learning, a further element may be added which stimulates the classroom concept – the dissemination of thoughts, ideas, knowledge, and team work between students during the learning process (13). According to this, peer learning has proved to be important for education, since it promotes communicative abilities, critical thinking, and self-confidence (14). Since aspects of peer learning are being incorporated into modern uses of e-learning (15), we were interested in exploring its potential value in a simple yet effective e-learning setup.

We suggest that an important factor may be social interaction modules like Internet forums. Although these have been used since the early 90s, the uses and potential in medical education have not been investigated properly. It seems that it is generally accepted that forums are reviewed positively by the students (16) and that it may have an impact on team working abilities and other non-clinical skills (17). However, it is not shown whether student's interactions facilitate better short-term learning strategies or better long-term retention of knowledge even though this is what they may feel (18).

## Aim

Since previous studies have compared mixed *levels* of learning methods to each other, our aim in this study was to compare different *levels* of e-learning to each other (level 1 vs. level 2). To verify that an improvement in levels does in fact improve learning ability, we did the same test for two different *types* of learning. Because of the production cost, we chose not to produce complex animation or high-fidelity/3D graphics limiting the study to *levels* 1 and 2.

The second aim was to evaluate if the social aspect would improve learning outcome. We created a third group (level 2+) equal to level 2 but with the possibility to interact in blog forums and in program messaging and compared this to the level 2 group.

## Materials and methods

### Learning courses and groups

Test subjects were Danish medical students in their third to fourth year of training. Students were recruited using a Facebook advertisement and using the “first come, first served” principle. The enrollment was closed after six groups of 20 students each were established. Each student was randomized to one of these groups. Before starting this course, students were given 2 weeks to take a pre-test of basic points that were to be included in the course itself. No students were admitted to the course without prior completion of the pre-test, but the results of the pre-test did not influence participation in the course. None of the students had prior experience working in the emergency room or at neurological departments. Group 1 (Group 1a [eCases level 1, *n*=20], Group 1b [eCases level 2, *n*=20], and Group 1c (eCases level 2+, *n*=20]) participated in an interactive case-based e-learning program, while Group 2 (Group 2a [eTextbook level 1, *n*=20], Group 2b [eTextbook level 2, *n*=20], and Group 2c [eTextbook level 2+, *n*=20]) were presented with textbook material electronically. The subject was head injury and associated treatment and observation guidelines in the emergency room. The content of Group 1 was based on two case stories and a description of one procedural skill, namely fluency in the Glasgow Coma Scale (GCS) score. Both groups were free to use the learning module as much as they desired. The students were asked to complete the post-test after finishing the e-learning module and were required to deliver it within 1 week. The improvement of each participant (post-test result minus pre-test result) was used as a statistic for comparison between groups. The learning objective included recall of the elements of the GCS (19) and analysis and application of knowledge to head trauma scenarios with the potential for complex physiological interactions and multi-organ involvement.

### Module setup

The pre-test–post-test, questionnaire and e-learning modules were all designed using Moodle – a free, open-source PHP web application for producing modular internet-based courses (also used in many universities – Minnesota, Cambridge, Oxford, Edinburgh, Washington, York, etc.) integrated into www.medviden.dk, a free Danish homepage for medical education. All parts of the study were closed and required a password for admittance, but in the future the e-learning modules will be opened for free access. The program was carried out in Danish.

### Pre- and post-tests

Pre- and post-tests both consisted of 25 questions randomly chosen from a pool of 50 different questions. To avoid confounding, the questions were shuffled for the two groups. The questions were provided in several formats including multiple choice (single best answer from multiple answers) and true/false questions. The questions included clinical photos, and both tests were based on true clinical stories. The questions were all used in former examinations at fifth or sixth years at Copenhagen University Faculty of Medicine thereby also verified by leading physicians. Both tests were reviewed online (pilot-tested) and validated by two senior doctors working with extensive experience in emergency medicine in Denmark securing the clinical relevance. The questions were rated as being of equivalent difficulty and of similar clinical relevance, and they ensured that the material covered by the tests was addressed by both the e-learning modules and the keynote presentation. Both tests were produced after the learning material to avoid the risk of teaching to the tests. The students had a time limit of 30 minutes for completion of each test. Only one correct answer was permitted for each question.

### eCases (Group 1)

The eCases module was prepared using clinical cases, pictures, and explanations. *Level 1*: A keynote presentation was uploaded, and the students had the possibility to read the slides more than once. Two case stories were presented, and the student was able to follow more than one path toward the conclusion of the case. *Level 2:* Instead of the keynote presentation, each slide was individualized, and part of the presentation was shown as a video with voice-over. Simple animations and short clinical videos were made and added to the case section and the student was asked to answer guided test questions before moving on to the two cases. *Level 2+* only differed from *level 2* by the social aspect. The student was able to see the other students’ pre- and post-test and could communicate freely on internal mails and blog forum.

### eTextbook (Group 2)

The eTextbook was an ordinary homepage presenting textbook material electronically. *Level 1*: Pure text with pictures and drawings and template-based presentation. *Level 2:* Video and simple animations were added. Each slide was individualized, and part of the presentation was shown as a video with voice-over. *Level 2+* only differed from *level 2* by the social interaction aspect. The student was able to see the other students’ pre-and post-test and could communicate freely on internal mails and blog forum.

### Questionnaire

All students participated in a questionnaire after completing the post-test. The questionnaire obtained general feedback regarding the educational method. A PDF version of the questionnaire is available [in Danish] upon request.

### Study size and statistical tests

Deriving experiences from a pilot study, the post-test–pre-test difference of positive answers in Group 2 (eTextbook) was anticipated to be 30% (an improvement in correct answers from 30 to 60%). Anticipated range for this difference would be 20–40%, thereby applying a standard deviation for all groups at 5%. Our anticipation was that all educational methods would only deviate slightly from each other, and a 5% difference was chosen as a minimum relevant difference (MIREDIF). Significance level was set at 5%, and statistical power at 80%. This yielded a total of 16 subjects in each group (http://www.opengcp.dk/calmiredif.php). To avoid an impact due to dropouts or missing data, it was decided that each group consisted of 20 subjects. Post-test–pre-test difference for each participant was chosen as our primary statistic. Given the limited number of participants, the Mann–Whitney *U* test was chosen for between-group comparisons. Cohen's *d* test was used to evaluate effect size.

## Ethics

The study was purely educational and the Danish National Committee on Health Research Ethics (DNVK), Regional Region was consulted. Their conclusion was that the study did not require ethical approval (h-4-2013-fsp 41).

## Results

All 120 students concluded both pre- and post-tests and the educational elements of the study. Pre-test and post-test results are presented in Table 2, with between-level comparisons in Table 3. Effect sizes are presented as Cohen's *d* and support a major improvement from pre- to post-test results.

**Table 2:** Correct answers in pre-tests and post-tests

Group	Pre-test	Post-test	Improvement	Cohen's *d*
1a (eCases, level 1)	22% (21–23%)	88% (86–91%)	66% (63–69%)	14.23
1b (eCases, level 2)	22% (20–23%)	92% (91–94%)	70% (68–73%)	19.78
1c (eCases, level 2+)	22% (21–23%)	94% (92–95%)	71% (68–73%)	18.24
2a (eTextbook, level1)	23% (21–24%)	66% (64–68%)	43% (41–45%)	10.51
2b (eTextbook, level 2)	22% (21–24%)	70% (68–73%)	48% (46–50%)	10.76
2c (eTextbook, level 2+)	22% (20–23%)	71% (68–74%)	49% (46–53%)	9.71

Values are relative numbers of correct answers for each type of educational content in each group, with 95% confidence intervals in brackets. *p* <0.05 for all of the following comparisons: 1a (eCases) vs. 2a (eTextbook); 1b (eCases) vs. 2b (eTextbook); 1c (eCases) vs. 2c (eTextbook). Mann–Whitney *U* test for all comparisons. *N*=20 in each group. Cohen's effect size value (*d*): high (>0.8), moderate (0.5–0.8), or small (0.2–0.5) practical significance.

**Table 3:** Between-level comparisons in test result improvements

Level comparisons	Results	*p*	Cohen's *d*
*Group 1 (eCases)*			
Level 1 vs. level 2	66% vs. 70%	0.03	0.86
Level 2 vs. level 2+	70% vs. 71%	0.73	0.07
*Group 2 (eTextbook)*			
Level 1 vs. level 2	43% vs. 48%	0.002	0.94
Level 2 vs. level 2+	48% vs. 49%	0.81	0.12

Mann–Whitney *U* test for all comparisons. *N*=20 in each group. Cohen's effect size value (*d*): high (>0.8), moderate (0.5–0.8), or small (0.2–0.5) practical significance.

The groups (and levels) have comparable pre-test results with 20–24% correct answers (Table 2).

### Levels

Students at level 2 (in both groups) improved statistically significant more than students at level 1 (Group 1a vs. Group 1b *p*=0.03; Group 2a vs. Group 2b *p*=0.002; Table 3). Cohen's *d* is high regarding the comparison between level 1 and level 2, but low between level 2 and level 2+.

In both groups, there was also statistically significant better satisfaction with the material (Group 1a vs. Group 1b *p*<0.001; Group 2a vs. Group 2b *p*=0.002; high effect sizes, Table 4), while satisfaction with the social aspect was equal.

**Table 4:** Student's satisfaction with the e-learning material and social aspects (score 1–10)

Level comparisons	Group 1	*p*	Cohen's d	Group 2	*p*	Cohen's *d*
*Satisfaction with material*						
Level 1 vs. level 2	4.2 vs. 6.9	<0.001	3.19	3.8 vs. 5.5	0.002	1.39
Level 2 vs. level 2+	6.9 vs. 7.0	0.94	0.06	5.5 vs. 5.7	0.76	0.08
*Satisfaction with social aspect*						
Level 1 vs. level 2	4.7 vs. 4.9	0.52	0.25	4.5 vs. 4.2	0.40	0.26
Level 2 vs. level 2+	4.9 vs. 6.9	<0.001	2.80	4.2 vs. 5.0	0.04	0.72

Arithmetic averages for each group displayed. Mann–Whitney *U* test for all comparisons. *N*=20 in each group. Cohen's effect size value (*d*): high (>0.8), moderate (0.5–0.8), or small (0.2–0.5) practical significance.

### Peer learning

There was no statistically significant difference between level 2 and level 2+ (Group 1b vs. Group 1c *p*=0.73; Group 2b vs. Group 2c *p*=0.81) when looking at improvement in learning. However, level 2+ was associated with statistically significant greater student satisfaction due to the social aspect than the rest of the students (Group 1b vs. Group 1c *p*<0.001; Group 2b vs. Group 2c *p*=0.04; high effect sizes, Table 4).

## Discussion

### Benefit of high-level e-learning

As expected, all students achieved statistically significant improvements in their post-test scores compared to the pre-test scores and the degree of improvement was dependent on the type of learning. The post-test improvements seen in Group 1 were statistically significant better than the improvements in Group 2 for each level of education (Table 2; *p*<0.05). Moreover, the degree of improvement was statistically significant higher for level 2 than level 1 educational material in both groups (Table 3; *p*<0.05). To our knowledge, this improvement has not been documented before, and the results emphasize the importance of a standardized description methodology in e-learning studies. It also highlights the potential effects of higher level e-learning. Former studies have shown that e-learning is at least as good as conventional learning (3), but a new look at the efficacy of different levels of e-learning is highly warranted.

### Benefit of social interaction

The value of level 2 e-learning with the possibility of peer learning deserves some discussion. While students performed equally well in the level 2 and level 2+ groups in terms of test results, we were able to identify considerable differences in student satisfaction, within both types of e-learning (Table 4; *p*<0.05). Our results suggest that Group 1 seemed to have the most benefit of the possibility to interact with other students (*p*<0.001). This social interaction, which may well correspond to classroom scenarios does not influence the students’ final improvements. However, little is known about the true value of this peer learning possibility because it may have helped facilitate better short-term learning strategies for the students, and may also improve the aspect of story-telling between students into the framework of the education, enhancing better long-term retention of learned material. We concede that the current study is not designed to investigate this aspect, but we suggest that future studies should dig deeper into this particular aspect of e-learning, since modern e-learning technology may adequately facilitate even complex social interaction between students. In addition, we are confident that the profuse dissemination and frequent use of internet-based social platforms will automatically add to the e-learning experience, whether we as teachers incorporate it into the module itself or not. There may be a bias regarding this particular part because the students were in fact recruited using a social medium, but we argue that the use of these social media are expending worldwide making this bias a more theoretical than actual nature.

### Benefits and resources

It is important to choose the right educational method to the content and purpose of the educational material. e-Learning has some benefits, while traditional teaching has others. e-Learning platforms are gradually getting ready to facilitate learning the way traditional teaching does when addressing the social aspect and the benefits of live classroom interaction (5, 6). e-Learning has potential advantages over didactic learning, both when looking at accessibility and advanced contents (multimedia and interactive navigation). This study has investigated simple to medium advanced e-learning tools, but we hypothesize that e-learning beyond level 2 may lead to competitions and direct student's interactions which may eventually resemble those seen in classroom settings (1). In our study, we discovered that that addition of a web forum for students was easy to implement and was associated with considerable satisfaction for the students. The ways to simple implementation of complex interactive materials is pivotal for the success of higher level e-learning (20). That being said, our study does not compare development costs to learning potential, but in this case the development time used by the teachers creating the theoretical part of the e-learning was roughly 50% longer when creating level 2. When looking at the additional programming and visual part, even more time was used. We do not believe the additional time used is comparable from course to course, since it depends not only on the educational level but also on how much of the material consists of video or animations, and how many resources are mandatory for the design element of the materials.

### Future medical education

Wutoh concluded in his review from 2004 that there is no significant difference between e-learning and didactic medical teaching (21). In the years since that review, the dramatic developments in e-learning contents and levels may herald an era of improved e-learning capabilities. At the very least, e-learning is as effective as traditional learning. This does not diminish the value of other skills, since good doctors require a diverse combination of skills, such as readily available knowledge, manual dexterity, clinical experience, and cognitive abilities. Although previous studies suggest that the educational need of this palette of skills may currently be difficult to satisfy with e-learning alone, future technological developments catering to such complex skill sets may not be far off with the increasing use of computer games, virtual reality simulations, and social networks (22). While the effective use of socially valuable information seems counterintuitive in e-learning platforms, our current study does argue that it is indeed possible to simulate peer learning aspects of classroom conditions in a simple and effective way, and that this interaction between students has some merits.

## Conclusions

This study underlines the importance of using a standardized and differentiated methodology when evaluating e-learning. Higher levels of e-learning does in fact provide better results when compared with the same type of e-learning at lower levels. We have questioned whether high levels would compare to even higher levels of education, and inquiries into the social aspects of learning between students seem to be the next logical step to improve the learning process. However inspiring these results may be, increased student satisfaction by the social interaction is offset by a failure to document an effect of this interaction on post-test improvements. We propose that future studies investigate whether student interactions may facilitate better short-term learning strategies or better long-term retention of student knowledge, and whether benefits of peer learning may be as significant in e-learning as in classroom or team-based teaching.

## References

[1] Kim S, Song SM, Yoon YI (2011). Smart learning services based on smart cloud computing. Sensors (Basel).

[2] Alur P, Fatima K, Joseph R (2002). Medical teaching websites: do they reflect the learning paradigm?. Med Teach.

[3] Cook DA, Levinson AJ, Garside S, Dupras DM, Erwin PJ, Montori VM (2008). Internet-based learning in the health professions. A meta-analysis. JAMA.

[4] Boulos MN, Maramba I, Wheeler S (2006). Wikis, blogs and podcasts: a new generation of web-based tools for virtual collaborative clinical practice and education. BMC Med Educ.

[5] Lam P, Au Yeung M, Cheung E, McNaught C (2009). Using the development of eLearning material as challenging and authentic learning experiences for students. Same Places, Different Spaces; Proceedings Ascilite Auckland 2009.

[6] Qi B, Liu L, Wang C (2009). E-learning tools to improve students’ learning experience: a case study. IJMECS.

[7] Bloom BS, Engelhart MD, Furst EJ, Hill WH, Krathwohl DR (1956). Taxonomy of educational objectives: the classification of educational goals; handbook 1: cognitive domain.

[8] Moule P, Albarran JW, Bessant E, Brownfield C, Pollock J (2008). A non-randomized comparison of e-learning and classroom delivery of basic life support with automated external defibrillator use: a pilot study. Int J Nurs Pract.

[9] Grijpink-van den Biggelaar K, Drop SL, Schuwirth L (2010). Development of an e-learning portal for pediatric endocrinology: educational considerations. Horm Res Paediatr.

[10] Mazzoleni MC, Rognoni C, Finozzi E, Gri T, Pagani M, Imbriani M (2011). E-learning for occupational physicians’ CME: a study case. Stud Health Technol Inform.

[11] Evgeniou E, Loizou P (2012). The theoretical base of e-learning and its role in surgical education. Surg Endosc.

[12] Kavadella A, Kossioni AE, Tsiklasis K, Cowpe J, Bullock A, Barnes E (2013). Recommendations for the development of e-modules for the continuing professional development of European dentists. Eur J Dent Educ.

[13] Ofstad W, Brunner LJ (2013). Team-based learning in pharmacy education. Am J Pharmaceut Educ.

[14] Stone R, Cooper S, Cant R (2013). The value of peer learning in undergraduate nursing education: a systematic review. ISRN Nurs.

[15] Sander B, Golas MM (2013). HistoViewer: an interactive e-learning platform facilitating group and peer group learning. Anat Sci Educ.

[16] Sucha M, Engelhardt S, Sarikas A (2013). Internet discussion forums as part of a student-centred teaching concept of pharmacology. GMS Z Med Ausbild.

[17] Choudhury B, Gouldsborough I (2012). The use of electronic media to develop transferable skills in science students studying anatomy. Anat Sci Educ.

[18] Carroll C, Booth A, Papaioannou D, Sutton A, Wong R (2009). UK health-care professionals’ experience of on-line learning techniques: a systematic review of qualitative data. J Contin Educ Health Prof.

[19] Teasdale G, Jennett B (1974). Assessment of coma and impaired consciousness: a practical scale. Lancet.

[20] Fox S, MacKeogh K (2003). Can eLearning promote higher-order learning without tutor overload?. Open Learning.

[21] Wutoh R, Boren SA, Balas EA (2004). ELearning: a review of internet-based continuing medical education. J Contin Educ Health Prof.

[22] Otero WRI, Petch JR, Catapan AH (2012). Developing high-level cognitive skills in e-learning. Rev Cientif Int.

